# How safe is teaching of highly specialized rectal surgery? A propensity score–matched 10‐year cohort study

**DOI:** 10.1111/codi.70544

**Published:** 2026-07-07

**Authors:** Simon Cheseaux, Luka Medic, Fabio Butti, Martin Hübner, Dieter Hahnloser, Amaniel Kefleyesus, Fabian Grass

**Affiliations:** ^1^ Department of Visceral Surgery Lausanne University Hospital CHUV, University of Lausanne (UNIL) Switzerland

**Keywords:** colorectal surgery, intraoperative adverse events, postoperative complications, propensity score‐matched analysis, teaching

## Abstract

**Aim:**

This study aimed to assess the impact of an institutional teaching program of highly specialized rectal procedures on intra‐ and early postoperative outcomes.

**Methods:**

We performed a retrospective 10‐year cohort study of consecutive patients undergoing low anterior resection (LAR) or abdominoperineal resection (APR) for rectal cancer <12 cm from the anal verge or restorative proctocolectomy with ileal pouch–anal anastomosis (IPAA) at a tertiary high‐volume academic centre (2014–2023). Procedures were classified as expert (consultant‐only) or teaching procedures (performed ≥75% by the trainee under direct supervision). Groups were balanced after 1:1 propensity score matching (PSM) for age, sex, BMI, ASA, Charlson index, neoadjuvant therapy and surgical approach. Primary outcomes were intraoperative surgical adverse events (iAEs, defined according to the Classintra classification) and 30‐day complications. Multivariable logistic regression identified predictors of morbidity.

**Results:**

A total of 573 surgeries were included (375 expert‐led, 198 teaching procedures). After matching, 374 remained (187 per group). IAEs occurred in 16% and 17% of expert‐led and teaching procedures, respectively (*p* = 0.9). Overall morbidity was 43% vs. 46% (*p* = 0.7); severe complications (Clavien–Dindo ≥ IIIb) occurred in 16% vs. 17% (*p* = 0.9), while 30‐day mortality was 1.1% vs. 0% (*p* = 0.5). Median length of stay was 8 vs. 7 days (*p* = 0.6). Reoperation within 30 days occurred in 15.5% in both groups, and readmissions in 16% vs. 10% (*p* = 0.2). Multivariable analysis revealed high comorbidity indices and immunosuppression as independent risk factors. Robotic surgery was independently associated with lower postoperative morbidity, while teaching was neither associated with overall morbidity (OR 0.60, 95% CI 0.30–1.17) nor severe complications (OR 0.64, 95% CI 0.26–1.48).

**Conclusion:**

Closely supervised teaching of highly specialized rectal surgery can be safely implemented into clinical practice without increasing perioperative morbidity, supporting the dual mission of surgical proficiency and training of high‐volume centres.

**What does this paper add to the literature?**

Centralization of complex rectal surgery to specialized centres provides opportunities for supervised training. However, the impact of surgical teaching on outcomes in this highly specialized setting remains uncertain. Therefore, this study aimed to assess the effect of trainee involvement on intraoperative and short‐term postoperative outcomes.

## INTRODUCTION

Rising healthcare costs and the well‐established correlation between surgical volume and improved outcomes have led to the centralization of highly specialized medical (HSM) procedures, including deep pelvic rectal resections, within academic centres [1]. Complex rectal surgery is classified as HSM in Switzerland, a mandate awarded to specific hospitals by a national decision‐making body [2]. This strategy aims to increase and concentrate institutional caseloads in high‐volume facilities in order to improve outcomes and foster supervised surgical training programs [3]. Furthermore, the training of the next generation of colorectal surgeons remains a core mission of high‐volume teaching facilities to ensure continuity of surgical care.

In the field of colorectal surgery, HSM procedures encompass low rectal cancer resections (LAR) and abdominoperineal resections (APR) for rectal cancer with a distal margin located within 12 cm from the anal verge (AV), anal cancer and restorative proctocolectomies with ileal pouch–anal anastomosis (IPAA) for ulcerative colitis (UC) or familial adenomatous polyposis (FAP) [4].

Several groups have investigated the effect of supervised surgical teaching on outcomes in colorectal surgery. While most focused on cancer resections, few addressed inflammatory bowel disease (IBD) surgery and restorative proctocolectomy with IPAA. Regarding rectal cancer surgery, a meta‐analysis reported no differences in anastomotic leak and conversion rates, R0 resection or local recurrence between teaching and non‐teaching cases [5]. Similarly, a Mayo Clinic study found no negative effect of trainee participation on oncologic indicators in robotic rectal cancer surgery, nor any significant prolongation of operative time [6]. Odermatt et al. confirmed these findings in a propensity score‐matched analysis, showing no differences in operative time, blood loss, conversion rate or complications in laparoscopic colorectal cancer resections [7].

However, to date, no analysis exclusively focused on highly specialized rectal procedures as defined above. This gap is relevant given the technical complexity and potential risks and challenges associated with highly specialized rectal procedures, where the effects of trainee involvement on perioperative and functional outcomes remain unclear. Furthermore, hands‐on training needed to develop the required surgical skills is acquired through substantial learning curves [8, 9].

The present study aimed to analyze the impact of an institutional teaching program of HSM rectal procedures on intra‐ and early postoperative outcomes.

## METHODS

This retrospective, single‐centre cohort study was conducted at the Department of Visceral Surgery, Lausanne University Hospital (CHUV). All adult patients with documented general consent who underwent HSM rectal surgery over a 10‐year period between 1 April 2014 and 31 December 2023, were included. The study was approved by the local ethics committee (Commission cantonale d'éthique de la recherche sur l'être humain, CER‐VD 2024‐01803; approval date November 2, 2024).

### Patients

Included were consecutive elective rectal HSM procedures including low anterior resections (LAR) and abdominoperineal resections (APR) for rectal or anal cancer (as per HSM definition of lower border within 12 cm of the anal margin as assessed by rigid rectoscopy) and restorative proctocolectomies with ileal pouch–anal anastomosis (IPAA) for UC or FAP. Open, standard laparoscopic and robotic approaches were considered. Minimally invasive procedures (laparoscopic or robotic) converted to open laparotomy were classified as either pre‐emptive (unrelated to an intraoperative adverse event, iAE) or reactive (in response to an iAE). Emergency procedures (surgery performed during non‐planned admission) were excluded.

Data were extracted from the prospectively maintained institutional database and managed by two dedicated data managers with regular audits by the consultant surgeons. Furthermore, database quality and completeness were cross checked yearly through official external audits according to Swiss HSM regulations [4].

Collected variables included demographic data (age, sex), preoperative status (WHO performance status, American Society of Anesthesiologists [ASA] score) and comorbidities as assessed by the Charlson Comorbidity Index [10]. Additional baseline factors were ongoing immunosuppression (i.e. steroids or biologic treatments) at the time of surgery, prior abdominal surgery and presence of active ulcerative colitis, defined as a total Mayo score ≥6 [11]. Disease‐specific variables included surgical indication (rectal/anal cancer vs. benign indications including active UC and FAP) and oncologic staging (TNM/UICC) for anal and rectal cancer [12]. Neoadjuvant therapy for rectal cancer (long‐course chemoradiotherapy (CRT), short‐course radiotherapy and/or neoadjuvant chemotherapy) were also recorded.

Surgical variables included procedure type, approach (open, laparoscopic, robotic, converted to open), operative time, estimated blood loss (calculated intraoperatively from suction canisters and sponges after subtracting irrigation fluids), transfusion requirements, contamination class [13], TME quality (complete, near complete or incomplete), completeness of resection (R0 vs. R1 vs. R2), height of the colorectal anastomosis (distance from AV) and creation of a protective ostomy (ileostomy or colostomy).

### Outcomes

Surgical iAEs were defined as any deviation from the ideal surgical course [14] and were classified according to ClassIntra [15]. The iAEs were bleeding above average that could require vessel ligation, suture or blood transfusion, and reactive conversion to open surgery. Peroperative transfusion included all intraoperative and 24‐h postoperative transfusions. The iAEs were also detailed in previous publications [16, 17]. Postoperative outcomes included length of stay, complications graded by the Clavien–Dindo classification (severe ≥IIIb) [18] and the comprehensive complication index (CCI) [19], surgical site infection (SSI) defined according to CDC criteria [20], anastomotic leak (clinically or radiologically confirmed), postoperative ileus and unplanned 30‐day readmission. Postoperative ileus was defined as intolerance of an oral diet with absence of flatus or stool by postoperative day 3–5, requiring radiologic evaluation or nasogastric decompression [21]. All patients were managed according to the institutional Enhanced Recovery After Surgery (ERAS) protocol [22].

### Definition of surgical teaching

Procedures were classified as expert (performed by a consultant colorectal surgeon) or teaching procedures (performed by a colorectal fellow or junior consultant under direct supervision during ≥75% of the operative time as defined by the HSM). All supervising consultants (DH, MH, FG) were board‐certified by the Swiss Society of Visceral Surgery and Fellows of the European Board of Surgery in Coloproctology (FEBS Coloproctology). Procedure classification (expert vs. teaching) was graded at the end of the operation by the trainee. In general, only senior fellows with advanced skills and experience were entrusted with teaching operations and in particular pelvic (TME) dissection. More junior fellows performed some of the routine steps (colonic mobilization, central vessel ligation, splenic flexure release) under direct supervision, but the procedures were then allocated to the expert group since not meeting the 75% target.

### Statistical analysis

Comparisons between teaching and non‐teaching procedures used propensity score methods to limit selection bias. Covariates were selected a priori based on clinical relevance and included age, sex, BMI, ASA class, Charlson Comorbidity Index (age‐adjusted), disease dignity (benign vs. malignant), preoperative radiotherapy for rectal cancer and surgical approach (open, laparoscopic, robotic). Patients were matched 1:1 using nearest neighbour matching without replacement.

Secondary analyses explored overall morbidity and severe complications using multivariable logistic regression within the matched cohort. Results were expressed as odds ratios (OR) with 95% confidence intervals and illustrated in forest plots.

All analyses were performed in R (R Foundation for Statistical Computing, Vienna, Austria). Propensity scores were estimated by logistic regression, with matching performed using the *MatchIt* package (nearest neighbour, no replacement, calliper = 0.2 SD of the logit of the propensity score). Covariate balance was evaluated with standardized mean differences (absolute SMD < 0.10 considered acceptable). Diagnostics (SMD tables, Love plots) were generated with the *cobalt* package (Figure S1). Figures were created with *ggplot2*, and regression outputs summarized using *broom*.

## RESULTS

### Patient characteristics

A total of 573 patients were included: 375 expert and 198 teaching procedures (Table 1). After 1:1 propensity score matching, 374 patients remained for analysis (187 per group). Baseline demographic and clinical characteristics were well balanced, with no significant differences between groups (all *p* > 0.2; Table 2).

**TABLE 1:** Baseline characteristics before matching.

Item	No teaching^1^ *N* = 375	Teaching^1^, * *N* = 198	Total^1^ *N* = 573	*p‐value* ^2^
Gender				
Female	145 (38.7)	97 (49.0)	242 (42.2)	
Male	230 (61.3)	101 (51.0)	331 (57.8)	0.022
Age, year (median, IQR)	59.7 (45.9 to 71.4)	62.1 (53.5 to 72.8)	61.0 (48.4 to 71.9)	0.051
BMI, kg/m^2^ (median, IQR)	24.3 (21.4 to 27.9)	24.7 (21.2 to 28.2)	24.4 (21.3 to 28.0)	0.787
ASA category				
≤2	268 (71.5)	139 (70.2)	407 (71.0)	
>2	107 (28.5)	59 (29.8)	166 (29.0)	0.825
Charlson				
≤2	220 (58.7)	104 (52.5)	324 (56.5)	
>2	155 (41.3)	94 (47.5)	249 (43.5)	0.186
Immunosuppression	42 (11.2)	25 (12.8)	67 (11.8)	0.689
Dignity of disease				
Benign	105 (28.0)	52 (26.3)	157 (27.4)	
Malignant	270 (72.0)	146 (73.7)	416 (72.6)	0.730
Previous abdominal surgery	200 (53.5)	113 (57.7)	313 (54.9)	0.388
Approach				
Laparoscopy	205	105	310	0.890
Open	94	43	137	
Robotic	76	40	116	
Active ulcerative colitis				
No	305 (81.3)	160 (80.8)	465	0.167
Yes	70 (18.7)	38 (19.2)	108	
Tumour stage (pre‐op)				
T0‐Tis	2 (1.1)	4 (3.7)	6 (2.1)	0.512
T1‐T2	23 (12.8)	15 (13.8)	38 (13.2)	
T2	6 (3.4)	4 (3.7)	10 (3.5)	
T3‐T4	148 (82.7)	86 (78.9)	234 (81.2)	
N0	85 (41.3)	44 (36.1)	129 (39.3)	0.452
N1	40 (19.4)	30 (24.6)	70 (21.3)	
N2‐N3	20 (9.7)	8 (6.6)	28 (8.5)	
Nx	61 (29.6)	40 (32.8)	101 (30.8)	
M0	161 (74.5)	97 (75.2)	258 (74.8)	0.994
M1	55 (25.5)	32 (24.8)	87 (25.2)	
NARTH				
None	57 (46.7)	47 (63.5)	104 (53.1)	0.027
Long‐course	40 (32.8)	21 (28.4)	61 (31.1)	
Short‐course	25 (20.5)	6 (8.1)	31 (15.8)	
NACT	32 (26.2)	19 (26.0)	51 (26.2)	1.000

Abbreviations: IBD, inflammatory bowel disease; IQR, interquartile range; NACT, neoadjuvant chemotherapy for rectal/anal cancer; NARTH, neoadjuvant radiotherapy for rectal/anal cancer.

* Procedure performed >75% under teaching supervision.

^1^ Median (IQR) for continuous variables; *n* (%) for categorical variables.

^2^ Wilcoxon rank sum test; Pearson's Chi‐squared test.

**TABLE 2:** Baseline characteristics after propensity score matching.

Item	No teaching^1^ *N* = 187	Teaching^1^, * *N* = 187	Total^1^ *N* = 374	*p‐value* ^2^
Gender				
Female	82 (43.9)	87 (46.5)	169 (45.2)	0.678
Male	105 (56.1)	100 (53.5)	205 (54.8)	
Age, year (median, IQR)	62.7 (48.0 to 72.2)	61.7 (52.0 to 71.9)	61.8 (49.9 to 72.1)	0.691
BMI, kg/m^2^ (median, IQR)	23.7 (21.1 to 27.1)	24.3 (21.2 to 27.8)	24.1 (21.2 to 27.6)	0.312
ASA				
≤2	130 (69.5)	135 (72.2)	265 (70.9)	0.649
>2	57 (30.5)	52 (27.8)	109 (29.1)	
Charlson				
≤2	107 (57.2)	100 (53.5)	207 (55.3)	0.533
>2	80 (42.8)	87 (46.5)	167 (44.7)	
Immunosuppression	28 (15.0)	26 (14.1)	54 (14.5)	0.917
Dignity of disease				
Benign	54 (28.9)	50 (26.7)	104 (27.8)	0.729
Malignant	133 (71.1)	137 (73.3)	270 (72.2)	
Previous abdominal surgery	88 (47.3)	104 (56.5)	192 (51.9)	0.095
Approach				
Laparoscopy	104 (55.6)	105 (56.1)	209 (55.9)	0.860
Open	50 (26.7)	46 (24.6)	96 (25.7)	
Robotic	33 (17.6)	36 (19.3)	69 (18.4)	
Active ulcerative colitis				
No	149 (79.7)	149 (79.7)	298 (79.7)	1.000
Yes	38 (20.3)	38 (20.3)	76 (20.3)	
Tumour stage (pre‐op)				
T0‐Tis	1 (1.0)	4 (3.8)	5 (2.4)	0.566
T1‐T2	17 (16.8)	15 (14.4)	32 (15.6)	
T2	3 (3.0)	4 (3.8)	7 (3.4)	
T3‐T4	80 (79.2)	81 (77.9)	161 (78.5)	
N0	56 (49.6)	45 (38.5)	101 (43.9)	0.273
N1	24 (21.2)	26 (22.2)	50 (21.7)	
N2‐N3	8 (7.1)	8 (6.8)	16 (7.0)	
Nx	25 (22.1)	38 (32.5)	63 (27.4)	
M0	94 (81.0)	95 (77.2)	189 (79.1)	0.574
M1	22 (19.0)	28 (22.8)	50 (20.9)	
NARTH				
None	38 (53.5)	44 (59.5)	82 (56.6)	0.741
Long‐course	22 (31.0)	21 (28.4)	43 (29.7)	
Short‐course	11 (15.5)	9 (12.2)	20 (13.8)	
NACT	12 (17.4)	16 (21.1)	28 (19.3)	0.728

* Procedure performed >75% under teaching supervision.

^1^ Median (IQR) for continuous variables; *n* (%) for categorical variables.

^2^ Wilcoxon rank sum test; Fisher's exact test; Pearson's chi‐squared test. IQR: interquartile range. IBD: inflammatory bowel disease. NARTH: neoadjuvant radiotherapy for rectal cancer. NACT: neoadjuvant chemotherapy for rectal/colon cancer.

### Intraoperative outcomes

Surgical approach distribution was comparable between the expert versus teaching group (respectively standard laparoscopy 55.6% vs. 56.1%, open 26.7% vs. 24.6%, robotic 17.6% vs. 19.3%). Median operative time was similar between groups (240 min [IQR 190–287] vs. 242 min [204–285]; *p* = 0.3). The overall rate of any iAEs was 16% in the expert group and 17% in the teaching group (*p* = 0.9). Estimated blood loss (median 100 mL in both groups; *p* = 0.8), peroperative transfusion requirement (8% vs. 7%; *p* = 0.6) and protective ostomy creation (66% vs. 60%; *p* = 0.2) did not differ significantly. Among conversions, 11.0% were pre‐emptive and 3.5% reactive, similarly distributed in both groups (*p* = 0.9). Surgical data is displayed in Table 3.

**TABLE 3:** Surgical data after propensity score matching.

Item	No teaching^1^ *N* = 187	Teaching^1^, * *N* = 187	Total^1^ *N* = 374	*p‐value* ^2^
Operative time, min				
Median (IQR)	240.0 (190.0 to 287.0)	242.0 (204.0 to 285.0)	241.0 (197.0 to 286.2)	0.262
iAEs	29 (16.0)	30 (16.7)	59 (16.3)	0.981
Intraoperative bleeding				
None	55 (29.4)	29 (15.9)	84 (22.8)	**0.010**
As usual	92 (49.2)	117 (64.3)	209 (56.6)	
More than usual, no relevant prolongation OR time	26 (13.9)	25 (13.7)	51 (13.8)	
More than usual, with significant prolongation OR time	14 (7.5)	11 (6.0)	25 (6.8)	
Estimated blood loss, mL				
Median (IQR)	100.0 (30.0 to 200.0)	100.0 (25.0 to 200.0)	100.0 (30.0 to 200.0)	0.793
Peroperative transfusion	15 (8.0)	12 (6.6)	27 (7.3)	0.733
Wound classification				
Clean	2 (1.1)	1 (0.5)	3 (0.8)	0.753
Clean‐contaminated	158 (84.5)	154 (83.7)	312 (84.1)	
Contaminated	23 (12.3)	22 (12.0)	45 (12.1)	
Infectious	4 (2.1)	7 (3.8)	11 (3.0)	
Type of rectal surgery				
HAR	27 (14.4)	35 (18.7)	62 (16.6)	0.507
LAR	137 (73.3)	128 (68.4)	265 (70.9)	
APR	23 (12.3)	24 (12.8)	47 (12.6)	
Conversion from MIS to open surgery	25 (13.4)	25 (13.4)	50 (13.4)	1.000
Conversion				
Pre‐emptive	19 (10.1)	21 (11.2)	41 (11.0)	0.889
Reactive	6 (3.2)	7 (3.7)	13 (3.5)	
Protective ostomy	124 (66.3)	112 (59.9)	236 (63.1)	0.238
Type of protective ostomy				
none	63 (39.0)	75 (44.3)	138 (41.6)	0.400
ileostomy	104 (61.0)	96 (55.7)	200 (58.4)	
Distance anastomosis from anal verge, cm				
Median (IQR)	2.5 (1.5 to 4.4)	4.0 (2.0 to 6.0)	3.0 (2.0 to 5.0)	**0.001**
TME quality				
complete	98 (77.2)	97 (72.9)	195 (75.0)	0.604
nearly complete	11 (8.7)	11 (8.3)	22 (8.5)	
incomplete	18 (14.2)	25 (18.8)	43 (16.5)	
R (resection)				
R0	101 (81.5)	117 (88.0)	218 (84.8)	0.334
R1	21 (16.9)	15 (11.3)	36 (14.0)	
R2	2 (1.6)	1 (0.8)	3 (1.2)	

*Note:* significant *p*‐values (*p* < 0.05) are highlighted in bold.

Abbreviations: APR, abdominoperineal resection; HAR, high anterior resection; IAE, intraoperative adverse event; IQR, interquartile range; LAR, low anterior resection; TME, total mesorectal excision.

* Procedure performed >75% under teaching supervision.

^1^ Median (IQR) for continuous variables; *n* (%) for categorical variables.

^2^ Wilcoxon rank sum test; Fisher's exact test; Pearson's chi‐squared test.

### Postoperative outcomes

30‐day complications are detailed in Table 4 and did not differ between the two groups.

**TABLE 4:** Postoperative outcomes after propensity score matching.

Complication	No teaching^1^ *N* = 187	Teaching^1^, * *N* = 187	Total^1^ *N* = 374	*p‐value* ^2^
Any complication	81 (43.3)	86 (46.0)	167 (44.7)	0.677
CCI				
Median (IQR)	0.0 (0.0 to 29.6)	0.0 (0.0 to 30.8)	0.0 (0.0 to 29.6)	0.934
Severe complications (Grade > 3a)	29 (15.5)	32 (17.2)	61 (16.4)	
30‐day mortality	2 (1.1)	0 (0.0)	2 (0.5)	
LOS, days				
Median (IQR)	8.0 (5.0 to 15.5)	7.0 (5.0 to 14.0)	7.0 (5.0 to 14.0)	0.584
Anastomotic leak	9 (4.8)	8 (4.3)	17 (4.6)	0.991
Wound infection (superficial SSI)	10 (5.3)	11 (5.9)	21 (5.6)	1.000
SSI	26 (13.9)	26 (13.9)	52 (13.9)	1.000
Bleeding	15 (8.0)	14 (7.5)	29 (7.8)	1.000
Ileus	38 (20.3)	37 (19.8)	75 (20.1)	1.000
30‐day reoperation	29 (15.5)	29 (15.5)	58 (15.5)	1.000
30‐day readmission	27 (16.0)	18 (10.3)	45 (13.1)	0.166

* Procedure performed >75% under teaching supervision.

^1^ Median (IQR) for continuous variables; *n* (%) for categorical variables.

^2^ Wilcoxon rank sum test; Fisher's exact test; Pearson's chi‐squared test. IQR: interquartile range. CCI: Comprehensive Complication Index. LOS: length of stay. SSI: surgical site infection.

### Multivariable analysis

**FIGURE 1:**
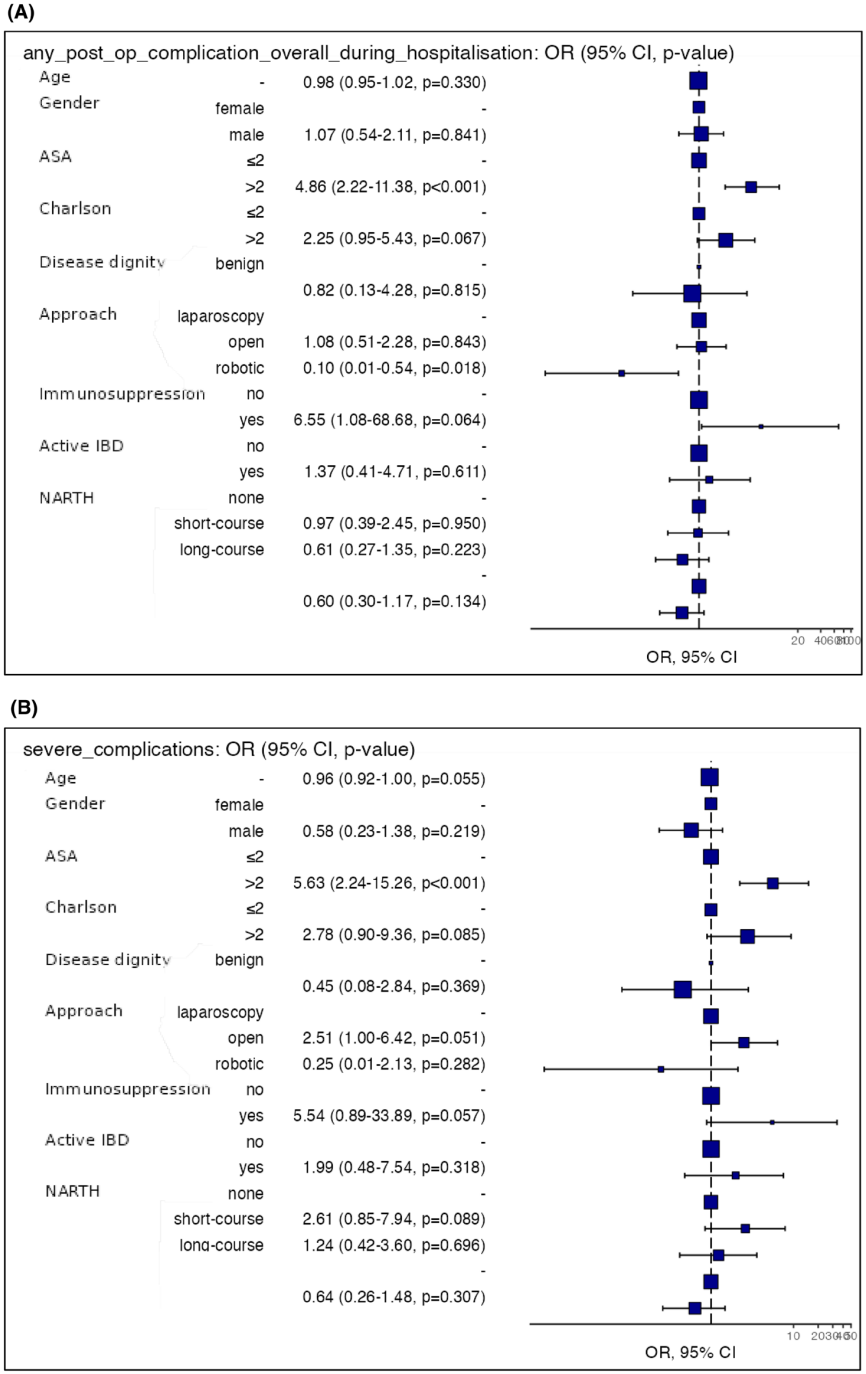
(A, B). Multivariable analysis displaying adjusted odds ratios (95% ci) for (A) overall postoperative complications and (B) severe complications in the matched cohort. Models adjust for age, sex, asa, charlson, dignity, surgical approach, immunosuppression, active ibd and preoperative radiotherapy; robust ses clustered by matched pair.

In adjusted logistic regression, teaching status was not independently associated with overall morbidity (OR 0.60, 95% CI 0.30–1.17; *p* = 0.134) nor severe complications (OR 0.64, 95% CI 0.26–1.48; *p* = 0.307). Independent risk factors of overall morbidity were ASA class >2 (OR 4.86, 95% CI 2.22–11.38; *p* < 0.001) and immunosuppression (OR 6.55, 95% CI 1.08–68.68; *p* = 0.064). Robotic approach was protective for overall morbidity (OR 0.10, 95% CI 0.01–0.54; *p* = 0.018). Severe complications were most strongly associated with ASA >2 (OR 5.63, 95% CI 2.24–15.26; *p* < 0.001), open surgery (OR 2.51, 95% CI 1.00–6.42; *p* = 0.051) and immunosuppression (OR 5.54, 95% CI 0.89–33.89; *p* = 0.057, Figure 1A, B).

### Sensitivity analyses

Findings remained consistent in sensitivity analyses stratified by surgical approach and when excluding conversions to open surgery.

## DISCUSSION

In this single‐centre PSM cohort of 573 consecutive patients undergoing complex rectal procedures, both intraoperative adverse events (iAEs) and short‐term postoperative outcomes were comparable between expert‐led surgeries and teaching operations performed under close supervision. Importantly, key indicators of surgical quality in cancer cases including the completeness of total mesorectal excision (TME) and R0 resection rates were no different. These findings demonstrate that teaching was safe in the demanding context of challenging pelvic surgery performed within the institutional HSM mandate in our high‐volume practice. This proof of safety is particularly relevant given the technical complexity of low pelvic dissection in the era of minimally invasive platforms.

Over the past three decades, minimally invasive techniques have been increasingly established. While laparoscopic surgery remains a standard for colonic surgery and high rectal tumours in most centres with limited access to the robotic platform, the latter is increasingly adopted for mid and low rectal tumours due to improved ergonomics, manoeuvrability and visualization in the narrow pelvis [23], resulting in reduced conversion rates and improved short‐term outcomes [24, 25]. Despite these assets, complication and readmission rates for highly specialized rectal surgery reach up to 50 and 25%, respectively, highlighting the challenges related to the surgical management of both low rectal cancer and UC patients [26]. Our analysis suggests a protective effect of robotics on postoperative outcomes, further supporting its systematic implementation in training and daily practice.

Previous work from our group has demonstrated that standardized intraoperative teaching protocols did neither increase intraoperative adverse events (iAEs) nor surgical site infections (SSIs) after colonic resections [16, 17]. Teaching is a central task of tertiary academic centres where complex disease presentations, advanced technology, and multidisciplinary strategies converge. Supervised, face‐to‐face and hands‐on operative training remains the cornerstone of subspecialty fellowship education. Systematic implementation of training opportunities into daily practice is essential to sustain high‐quality care. Centralization of HSM procedures within high‐volume centres is important for several reasons. First, iAEs negatively affect postoperative recovery and hospital stay; hence, training in high‐volume practices to improve surgical skills appears mandatory [27]. Second, high case volumes provide an ideal environment for surgical training at different levels without compromising outcomes [28]. Third, it was repeatedly shown that cumulative institutional experience translates into lower complication rates and improved results, specifically in rectal cancer surgery [29, 30]. Finally, multidisciplinary management in specialized tertiary centres improves long‐term oncologic outcomes, including reduced local recurrence and better survival [31]. This applies equally to complex benign conditions such as IBD and FAP, which also require highly coordinated specialist care [32].

This cohort included both anal and rectal cancer vs. IBD cases, two very heterogeneous populations with distinct but equally demanding features. In IBD, long‐term corticosteroid or immunosuppressive treatment may impair healing and increase septic risk, while younger patient age and the need to preserve long‐term function represent further challenges [33, 34]. Similarly, rectal cancer surgery also thrives to preserve patient function and quality of life. Similarly to the present results, a meta‐analysis demonstrated no difference in R0 resection, anastomotic leak and conversion rates, as well as operative time and blood loss between expert and teaching cases in rectal cancer surgery [5, 7]. Our present results thus further help to mitigate safety concerns related to surgical teaching, even in the setting of pelvic HSM procedures. Of note, the higher rate of intraoperative bleeding in expert‐performed procedures may be related to higher technical complexity as reflected by lower anastomoses in this group.

The absence of significant differences between expert‐ and trainee‐performed procedures in our study may be explained by the standardized framework of our institutional teaching program. Fellows performed most of the operation under constant supervision and were already highly experienced board‐certified general surgeons in training for sub‐specialization in visceral surgery or EBSQ coloproctology certification before being entrusted with HSM procedures and TME dissection. Finally, this mixed cohort (cancer and IBD) reflects real‐world HSM practice; future work may explore indication‐specific subgroup analyses.

## LIMITATIONS

This study has limitations related to its retrospective, single‐centre design with potential inherent selection bias. The heterogeneous group of HSM procedures was intentionally chosen to reflect real‐world data given all procedures included were performed within the same teaching setting. Recall bias was mitigated by systematic cross‐checks of the prospectively maintained database and regular audits by HSM authorities but cannot be excluded. External validity may be limited, as our centres' may not reflect practices elsewhere. The definition of teaching cases (≥75%) remains partly subjective, and brief expert interventions during difficult steps may introduce bias. The final decision whether to allocate an intervention to the teaching group was made by the trainee, to best reflect the trainees' perception. However, whether an intended teaching procedure was transformed into an expert procedure and how this potentially impacted on the results was not specifically assessable using this approach. Estimated intraoperative blood loss, although crosschecked with anesthesiologists, was surgeon‐reported and subject to measurement error. Despite rigorous adjustment through PSM, residual confounding from unmeasured variables cannot be excluded. Finally, long‐term functional and oncologic outcomes were not evaluated and will be the subject of future analyses. Moreover, subtle expertise‐related dimensions—such as stress management, operative strategy or technical finesse—were not captured in the setting of this study. Finally, the study was not designed for specific outcomes and may be underpowered for some of the rare adverse events. Hence, the results should be interpreted with caution.

Future research should focus on prospective multicentre studies with long‐term follow‐up, including functional and oncologic outcomes, to further validate the safety and educational value of surgical teaching of highly specialized rectal surgery.

## CONCLUSION

Closely supervised teaching of highly specialized rectal surgery can be safely implemented into clinical practice, with no increase in intraoperative or short‐term postoperative complications. High‐volume academic centres can thus ensure surgical proficiency while maintaining high‐quality surgical education.

## AUTHOR CONTRIBUTIONS

**Fabio Butti:** Data curation; resources. **Dieter Hahnloser:** Supervision; project administration; writing – review and editing. **Luka Medic:** Conceptualization. **Martin Hübner:** Project administration; supervision; writing – review and editing. **Amaniel Kefleyesus:** Methodology; investigation; software; data curation; validation; formal analysis; writing – review and editing. **Simon Cheseaux:** Writing – original draft; conceptualization; investigation; visualization. **Fabian Grass:** Conceptualization; methodology; data curation; investigation; validation; supervision; visualization; project administration; writing – review and editing; resources.

## FUNDING INFORMATION

The authors have nothing to report.

## ETHICS STATEMENT

The study was approved by the local ethics committee (Commission cantonale d'éthique de la recherche sur l'être humain, CER‐VD 2024‐01803; approval date November 2, 2024).

## CONFLICT OF INTEREST STATEMENT

The authors declare no conflicts of interest.

## Supporting information


**Figure S1.** Covariate balance‐Love plot of absolute standardized mean differences (SMDs) before and after 1:1 matching. The dashed line indicates SMD = 0.10.

## Data Availability

The data that support the findings of this study are available on request from the corresponding author. The data are not publicly available due to privacy or ethical restrictions.
