# Supplementary material for: How safe is teaching of highly specialized rectal surgery? A propensity score–matched 10‐year cohort study
Source: Colorectal Dis. 2026 Jul 7;28(7):e70544. doi: 10.1111/codi.70544 (PMC13342491; doi:10.1111/codi.70544)
Supplement: Supplementary file 1 — Figure S1. Covariate balance‐Love plot of absolute standardized mean differences (SMDs) before and after 1:1 matching. The dashed line indicates SMD = 0.10. [file CODI-28-0-s001.docx]

**SUPPLEMENTARY MATERIAL
Figure S1**: Covariate balance - Love plot of absolute standardized mean differences (SMDs) before and after 1:1 matching. The dashed line indicates SMD=0.10.


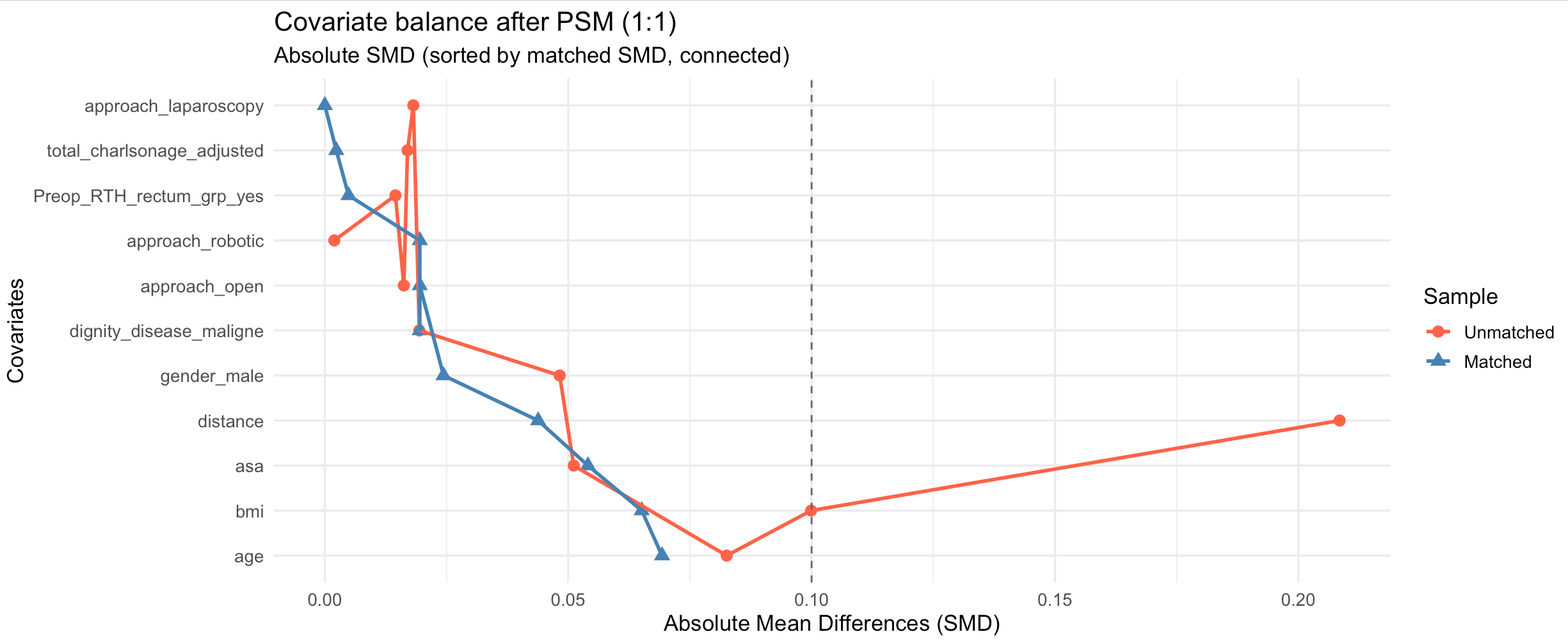


malignant
